# The effects of the PDE4 inhibitor roflumilast on cognitive performance after a cerebrovascular accident: a double-blind randomized placebo-controlled trial with an open label extension (ROSTMEMA)

**DOI:** 10.1177/02698811261436581

**Published:** 2026-04-24

**Authors:** Jill Kerckhoffs, Ieke Winkens, Arthur Ooghe, Samuel Branders, Jos Prickaerts, Arjan Blokland

**Affiliations:** 1 Department of Neuropsychology and Psychopharmacology, Faculty of Psychology and Neuroscience, Maastricht University, the Netherlands; 2 Limburg Brain Injury Centre, Maastricht University, the Netherlands; 3 Cognivia, Mont-Saint-Guibert, Belgium; 4 Peitho Translational, Maastricht, the Netherlands

**Keywords:** cerebrovascular accident, phosphodiesterase-4 inhibitor, roflumilast, cognition improvement, memory

## Abstract

**Background:**

Effective pharmacological treatments for Post-Stroke Cognitive Impairment (PSCI) remain elusive. Preclinical studies have shown that phosphodiesterase 4 (PDE4) inhibition improved cognition, particularly memory, in post-stroke animal models and in healthy young and elderly individuals. This study tested whether the PDE4 inhibitor roflumilast could improve memory in PSCI patients.

**Methods:**

A double-blind randomized placebo-controlled trial (RCT) included 100 community-dwelling participants receiving roflumilast (100 µg q.d.) or placebo (***N*** = 50 per group). Participants were 41–70 years and had suffered a cerebrovascular accident more than a year ago. They had subjective memory complaints and scored below the normative score on the delayed recall of the verbal learning test (VLT). After the RCT, 42 placebo group participants completed a 3-month open label extension (OLE). The primary outcome was the VLT delayed recall score. Roflumilast efficacy in the RCT was estimated through analysis of covariance (ANCOVA) with (post hoc) and without adjustment for baseline prognostic covariates defined a priori. In the OLE, general linear model repeated measures analyses were used. Secondary outcomes related to cognition, mood, and daily functioning.

**Results:**

Of the 97 participants completing the study (roflumilast: 48; placebo: 49), primary ANCOVA indicated a larger response in the roflumilast group on VLT and Rivermead Behavioural Memory Test (stories) at endpoint with non-significant moderate effect sizes (Cohen’s ***d***: 0.31–0.36). When post hoc adjusting for prognostic baseline covariates, effect sizes (0.33–0.40) became significant. Adverse events were similar in both groups. In the OLE, all memory test scores improved with medium to large significant effect sizes (Partial η^2^: 0.079–0.171).

**Conclusions:**

Roflumilast appeared to improve memory and was not associated with adverse effects. Results support further clinical studies.

## Introduction

The lifetime stroke risk has risen 50% in the past two decades, currently affecting one in four individuals worldwide (Feigin et al., 2022). Cognitive deficits are present in over 70% of stroke survivors (Rost et al., 2022). Post-Stroke Cognitive Impairment (PSCI) encompasses syndromes from mild cognitive impairment to dementia manifesting 3–6 months after a stroke and predominantly includes memory deficits, attentional deficits, and impaired executive function (Park et al., 2016; Rost et al., 2022). It imposes a substantial burden on healthcare systems, patients, and caregivers and notably impacts patients’ daily functioning (Rost et al., 2022).

Despite endeavors, effective pharmacological treatments for PSCI remain elusive (Huang et al., 2022). Enhancing synaptic plasticity is a potential target for drug treatment as it upregulates the processing and storage of information in the central nervous system, thereby improving cognitive functions (Murphy and Corbett, 2009). It is well established that the inhibition of phosphodiesterase type 4 (PDE4) has beneficial effects on neuronal signaling by enhancing cyclic adenosine monophosphate (cAMP) dependent long-term potentiation (LTP) (Blokland et al., 2019a; Reneerkens et al., 2009). LTP is assumed to be the physiological substrate of learning and memory and is associated with the formation of new synaptic connections (Bollen et al., 2014; Matsuzaki et al., 2004). Interestingly, phosphodiesterases (PDEs), including the subtype PDE4, are upregulated directly after brain injury (Wilson et al., 2016). This upregulation leads to enhanced degradation of cAMP and consequently may impair neuroplasticity. However, there are no experimental studies that have shown long-term upregulation of PDE4 after stroke (Ponsaerts et al., 2021). Irrespective of the degree to which PDE4 expression is altered 1 year after stroke, pharmacological PDE4 inhibition may nevertheless augment cAMP-mediated signaling and consequently improve memory function.

Along this line, post-stroke animal models have shown that behavioral and cognitive outcome was improved after treatment with PDE4 inhibitors, while central cell loss was prevented (Schreiber et al., 2020; Soares et al., 2015; Vilhena et al., 2021). The PDE4 inhibitor-induced increase in neuroplasticity was also observed in related animal brain injury models, including a study in which PDE4 inhibitors were administered 3 months after traumatic brain injury. The study showed that PDE4 inhibition reversed learning and memory deficits while also reversing the depression in LTP as well the loss of cells (Titus et al., 2018). In addition to enhanced neuroplasticity, anti-inflammatory mechanisms of action have been suggested for PDE4 inhibition (Schreiber et al., 2020; Soares et al., 2015), although this may be related to higher doses (Prickaerts et al., 2024).

PDE4 inhibitors have been shown to have beneficial effects on cognitive functions in Fragile X and schizophrenia patients (Berry-Kravis et al., 2021; Gilleen et al., 2021). However, the development of classic PDE4 inhibitors as therapeutic drugs has been hampered by their dose-limiting adverse effects, such as nausea and dizziness. Interestingly, the PDE4 inhibitor roflumilast, which has been approved as an anti-inflammatory drug for the treatment of chronic obstructive pulmonary disease (dose 250–500 µg), seems to induce fewer side effects. Previous studies with young and old healthy volunteers indicate limited side effects at acute doses of 250–300 µg as compared with a placebo. Interestingly, memory-enhancing effects were found at an acute dose of 100 µg, which was devoid of the typical side effects in these young and old healthy volunteers (Blokland et al., 2019b; Van Duinen et al., 2018). Given its favorable therapeutic window, roflumilast represents a compelling candidate for evaluating the therapeutic potential of PDE4 inhibition in patients with cerebrovascular accident (CVA)-related cognitive impairments (Prickaerts et al., 2024).

Therefore, the current study was designed to test whether PDE4 inhibition could be used to treat patients suffering from cognitive problems after CVA. In a double-blind randomized placebo-controlled trial (RCT), participants were included who suffered a CVA (all subtypes including transient ischemic attack and subarachnoid bleeding) at least 1 year ago, with subjective memory complaints and scored below the normative score on a word learning task. Since spontaneous recovery is no longer expected and cognition is not likely to improve 1 year after CVA, treatment effects can be investigated at this late stage after CVA (Rost et al., 2022). It was decided to include all subtypes, severities, and locations of CVA. This decision was made since the working mechanism of PDE4 inhibition to improve cognition does not depend on the type of CVA. A 100-µg dose of roflumilast was selected based on earlier acute studies demonstrating cognitive effects at this dose devoid of typical side effects in healthy participants (Blokland et al., 2019b; Van Duinen et al., 2018). At the time of study design, no data were available on chronic administration of roflumilast in post-stroke patients; therefore, this dose was chosen as a starting point expected to induce acute pharmacodynamic effects. After the RCT, the participants of the placebo group were given the opportunity to participate in a 3-month open label extension (OLE) study. This study aims to show the potential of PDE4 inhibition as a pharmacotherapeutic treatment to enhance cognition after CVA.

## Methods

### Study design

The main study was a double-blind, randomized placebo-controlled, between-subjects design and was a community-based parallel-group, two-arm (*N* = 50 per arm), superiority trial with a 1:1 allocation ratio. This was followed up by an OLE study. Recruitment was conducted at a single site: Maastricht University, the Netherlands. Recruitment for the trial took place over a 2-year period. The trial was registered with EudraCT (2020-003768-16**)** and Clinicaltrials.gov (NCT04854811).

All procedures were approved by the local Medical Ethics Committee (NL74897.068.20/METC20-068) and were conducted in accordance with the Helsinki Declaration of 1975 (as revised in 1983) and the Medical Research Involving Human Subjects Acts. This study was performed in accordance with the Consolidated Standards of Reporting Trials (CONSORT) (Cuschieri, 2019).

### Participants

Patients were recruited through social media and local caretaking organizations. Participants were eligible if they: had suffered a CVA (all subtypes including transient ischemic attack and subarachnoid bleedings) after the age of 40, and at least 1 year ago as diagnosed by a medical doctor; were between 41 and 70 years of age at the time of inclusion; had subjective memory complaints; and had a Verbal Learning Test (VLT) delayed recall score below the normative score (*z*-score below 0.00) corrected for gender, age, and education (Van der Elst et al., 2005). Of note, the z-score cut-off was originally set at between 1 and 3 standard deviations (SDs) below 0.00 but was adjusted through a protocol amendment to facilitate recruitment. The age group 41–70 years of age at the time of inclusion was chosen, since CVA’s before the age of 40 are frequently atypical and often stem from genetic predispositions or vascular malformations. These strokes are more diverse in their etiologies (Hussain et al., 2021). Additionally, above 70 years old, the risk of additional age-related problems or neurodegenerative diseases would increase. Subjects had to undergo a medical examination by a medical doctor. This included a medical questionnaire, blood and urine screening, and an electrocardiogram by a physician to exclude any underlying and potentially interfering pathologies. After approval of the research protocol, minor adaptations were made to the exclusion criteria (for a full overview, see Supplemental Appendix A). All participants gave written informed consent. Forty-four participants of the placebo group agreed to participate in a 3-month OLE study during which they received roflumilast treatment. Four participants did not want to continue due to the time investment and/or personal circumstances, and one participant no longer met the inclusion criteria. In both parts of the study, drop-outs were not replaced, as a 10% drop-out was taken into account for the sample size selection.

### Randomization and masking

Randomization and treatment allocation were performed by an independent person. Subjects were randomly assigned to the placebo or 100-μg roflumilast group. The allocation sequence was generated using a computerized random number generator that provided a seed (https://commentpicker.com/random-number-generator.php) and generated a ‘random’ order of two conditions in blocks of 20. This randomization was connected to participant numbers on the medication and communicated directly to the pharmacy and manufacturer of the capsules. Randomization was not stratified. The study researchers and the participants were blinded for allocation to the treatment group, throughout the entire treatment and assessment period of the RCT (not for the OLE). The authors were not blinded for treatment-group throughout the data analyses. Treatments were administered in identical capsules to ensure double blinding.

### Procedure

In the current study, only one daily dose strength of roflumilast (trade name: Daxas (EU), (Daliresp (US)) was compared to a placebo. A Good Manufacturing Practice-certified manufacturer (Basic Pharma Technologies BV, Geleen, the Netherlands) was appointed for ordering and reprocessing roflumilast tablets into capsules with our defined doses. Roflumilast tablets were crushed to a powder, and the powder was mixed with filler lactose monohydrate in the appropriate proportions. Identical size 0 capsules were manufactured with 0- and 100-μg roflumilast. These indistinguishable capsules were packaged in identical bottles and were sent to the hospital pharmacy. The pharmacy handed out these blinded and labeled bottles to the researchers after receiving the signed prescription from our medical doctor. The participants received a bottle with 50 capsules from the researchers twice (at the start of treatment and after 6 weeks at the second test session) and were instructed to take 1 capsule per day orally. For the OLE, the same dispensing procedure was followed, but participants received only unblinded bottles containing 100-μg roflumilast capsules.

**Figure 1.**
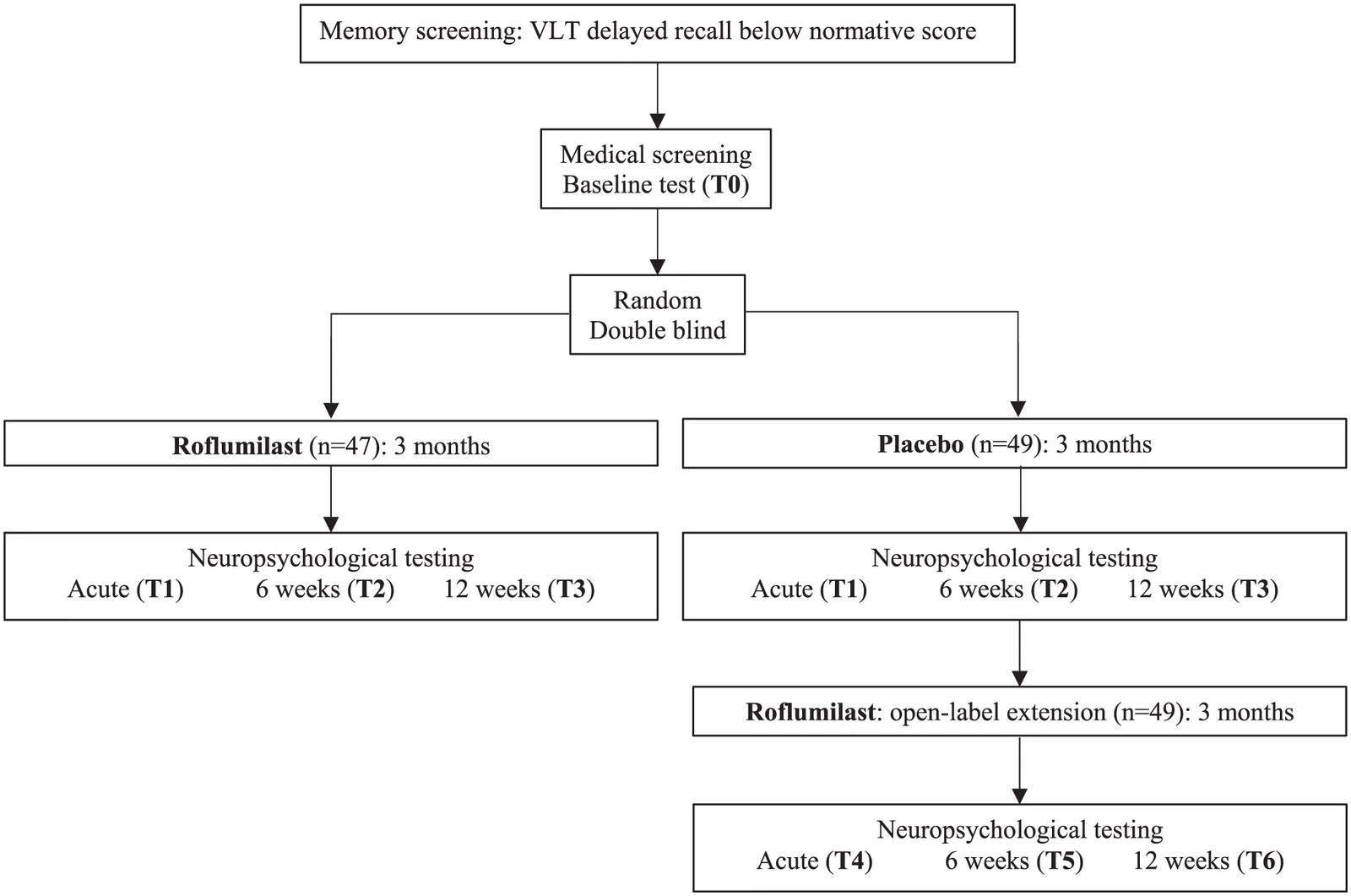
Flowchart of the experimental procedures. ***Note.*** T0–T6 refer to the different test sessions during the study. VLT: Verbal learning test, Normative score: corrected for gender, age, and education.

Participants came to the University once for screening and baseline testing (T0). All three follow-up meetings were at their homes. An overview of the measurements per time point is shown in Figure 1. The total duration of the treatment was 12 weeks. During the first measurement at home (T1), scheduled between 1 week and 3 months after T0, participants started treatment, and acute effects were measured 1 hour after the first drug intake. This is corresponding to the time of maximum plasma concentration (Tmax) of roflumilast, when peak plasma levels are reached and transient cognitive effects associated with maximal PDE4 inhibition are most likely to occur (Prickaerts et al., 2024). The next measurements were 6 weeks (T2) and 12 weeks (T3), respectively, after the start of the treatment. During the OLE, the participants were unblinded and tested at home for three more sessions at the same timepoints (T4–T6), starting at least 1 month after T3.

At all times, participants were instructed to refrain from consuming alcohol for 24 hours prior to each test session and to avoid caffeinated drinks and cigarettes on the morning of each session. They were reminded of this instruction the day before each assessment. However, compliance was not objectively verified, and therefore, we had to rely on participants’ adherence to these instructions. Participants kept a daily medication diary to monitor intake and side effects. No participants had to be excluded due to non-compliance. Although a few participants reported having missed an occasional dose, no one missed a significant number of doses, that is, more than 3 days, that would have affected treatment exposure during a part of the study. The number of capsules was checked each test session, to check adherence. All participants received financial compensation for follow-up measurements (€20 per session), and travel expenses for baseline testing were reimbursed.

During the 3-month treatment (in the RCT and the OLE), all participants performed a non-adaptive computerized test battery (Cogstate; https://www.cogstate.com/). The Cogstate Battery that was used included the following tests: the Groton Maze Learning Test (spatial executive functioning), the Detection Test (psychomotor function), the Identification Test (focused attention), the One Card Learning Test (visual learning), and the One Back Test (working memory). These tasks were different from the test battery that was used in the RCT/OLE. Therefore, no interference of tests was expected to occur. The Cogstate Battery was included to ensure that the participants were cognitively challenged during the treatment period to stimulate the efficiency of roflumilast and increase the likelihood of improving neuronal plasticity. These tests were conducted five times a week and each session took about 15 minutes.

### Outcomes

Demographical and CVA-related information were asked at baseline, including date of birth, gender (assessed with open-ended self-report question), level of education, date, and type of CVA, time spent in hospital, and rehabilitation. The primary outcome was verbal episodic memory as assessed with the delayed recall of the 15-word Verbal Learning Test (VLT) (Van der Elst et al., 2005). The VLT was used to assess short-term and delayed episodic memory. Based on earlier roflumilast research, main drug effects were expected on this cognitive function (Blokland et al., 2019b; Van Duinen et al., 2018). This VLT is a Dutch adaptation of the original 15-word Rey Auditory VLT (Rey, 1958). Normative scores of this test (based on age, education, and sex) are based on a large sample study (*n* = 1855) (Van der Elst et al., 2005). The VLT is a well-validated, reliable, and examiner-administered instrument widely used to measure verbal episodic memory (Possemis et al., 2024). In the VLT, 15 monosyllabic Dutch words are presented in five consecutive trials. For each test session, a different parallel version was used, which was equally difficult (unpublished data). The participants recalled the words immediately after each of the five trials (immediate recall), and this was then repeated five times. Words were shown for 2 seconds with a 1-second interval. After a 30-minute interval, during which they performed other tests, there was an additional free recall trial (delayed recall). Outcome scores were defined as the sum of the number of recalled items in the immediate trials, and the score on the delayed test. Immediate recall scores were included as secondary outcomes.

All secondary study parameters were administered at all time points. For measuring additional effects on everyday memory, the Rivermead Behavioural Memory Test (RBMT) subtest ‘stories’ was used to assess a person’s ability to absorb verbal information (Wilson et al., 1985). The RBMT is a functional memory assessment evaluating an individual’s ability to use memory function for the performance of daily tasks and attempts to bridge conventional and behavioral procedures. This test has demonstrated very high inter-rater reliability and good alternate form reliability. This allows for repeated assessments. Between test sessions, two equally difficult parallel test versions were alternated (Makatura et al., 1999).

Additionally, the Letter-Digit Substitution Test (LDST) and Trail Making Test (TMT) were used to test the effects on other cognitive functions (Reitan, 1986; Van der Elst et al., 2006). The LDST was used to assess a broad range of cognitive operations, such as sustained attention, psychomotor speed, visual scanning, mental flexibility, and speed of information processing (Van der Elst et al., 2006). The LDST is a paper-and-pencil cognitive test presented on a single sheet of paper that requires a subject to match letters to numbers according to a key located at the top of the page. In part one, the numbers are written down, and in part two, the numbers are read out loud; this is especially important to control for motor difficulties due to the CVA. The number of correct symbols within the allowed time of 90 seconds constitutes the score. For the LDST, two alternating parallel versions were used. Speed of processing, executive function, and mental flexibility were assessed by means of the TMT (Reitan, 1986). The test consists of two parts. Part A requires an individual to draw lines sequentially connecting 25 encircled numbers distributed on a sheet of paper. This part tests reaction time of psychomotor speed and visuospatial tracking. In part B, participants have to alternate between ordering the numbers and the alphabetical order of letters (e.g. 1, A, 2, B, 3, etc.), which tests cognitive flexibility and divided attention. The score on each part represents the amount of time required to complete the task. A ratio score (TMTB/A index) is calculated (i.e. time on card B divided by time on card A) and provides an indicator of executive functioning and mental flexibility (Jolles et al., 1995; Tombaugh, 2004).

To measure the more general and clinically relevant effects of roflumilast treatment on daily memory and activities of daily living and mood, the Everyday Memory Questionnaire-Revised (EMQ-R), the Utrecht Scale for Evaluation of Rehabilitation-Participation (USER-P), and the Hamilton Anxiety and Depression Scale, subscale Depression (HADS-D) were used (Post et al., 2012; Royle and Lincoln, 2008; Zigmond and Snaith, 1983). The EMQ-R was used to assess subjective memory complaints in daily living. The EMQ-R is a questionnaire that requires participants to respond to a list of memory-related behaviors by providing an estimate of how many times this happened to them over the previous month. Higher scores are indicative of a greater presence of memory difficulties. According to Royle and Lincoln (2008), it is a valid and reliable tool that has good face validity for use with neurological patients. Changes in behavior and participation in daily life were measured by the USER-P (Post et al., 2012). The USER-P is a generic measure of participation. It is divided into three scales: Frequency, Restrictions, and Satisfaction. The sum of scores for the scales is based on the items that apply to the patient’s situation and each sum score is converted to a 0–100 scale with higher scores indicating better participation (i.e. higher frequency, fewer restrictions, and higher satisfaction). The USER-P has adequate reliability and validity in patients with physical disabilities (Post et al., 2012). To assess changes in the participants’ mood, the HADS-D was used (Zigmond and Snaith, 1983). Bjelland et al. (2002) have concluded through a large review of studies that the identified cut-off point is 8 out of 21 for depression. The sensitivity and specificity of the HADS-D is sufficient (0.80).

Adverse events were monitored in medication diaries.

### Statistical analysis

The sample size calculation of 50 participants per group for the RCT was based on a previous study in which a positive effect of roflumilast on memory performance was found in the VLT (Blokland et al., 2019b). In the previous study with a healthy elderly population, an effect size (Cohen’s d) of 0.7 was found (Blokland et al., 2019b). Also, a power of 0.95 and α of 0.05 were used for G*power calculation. Based on these parameters, 45 participants per treatment group were needed. A drop-out of 10% was anticipated.

**Figure 2.**
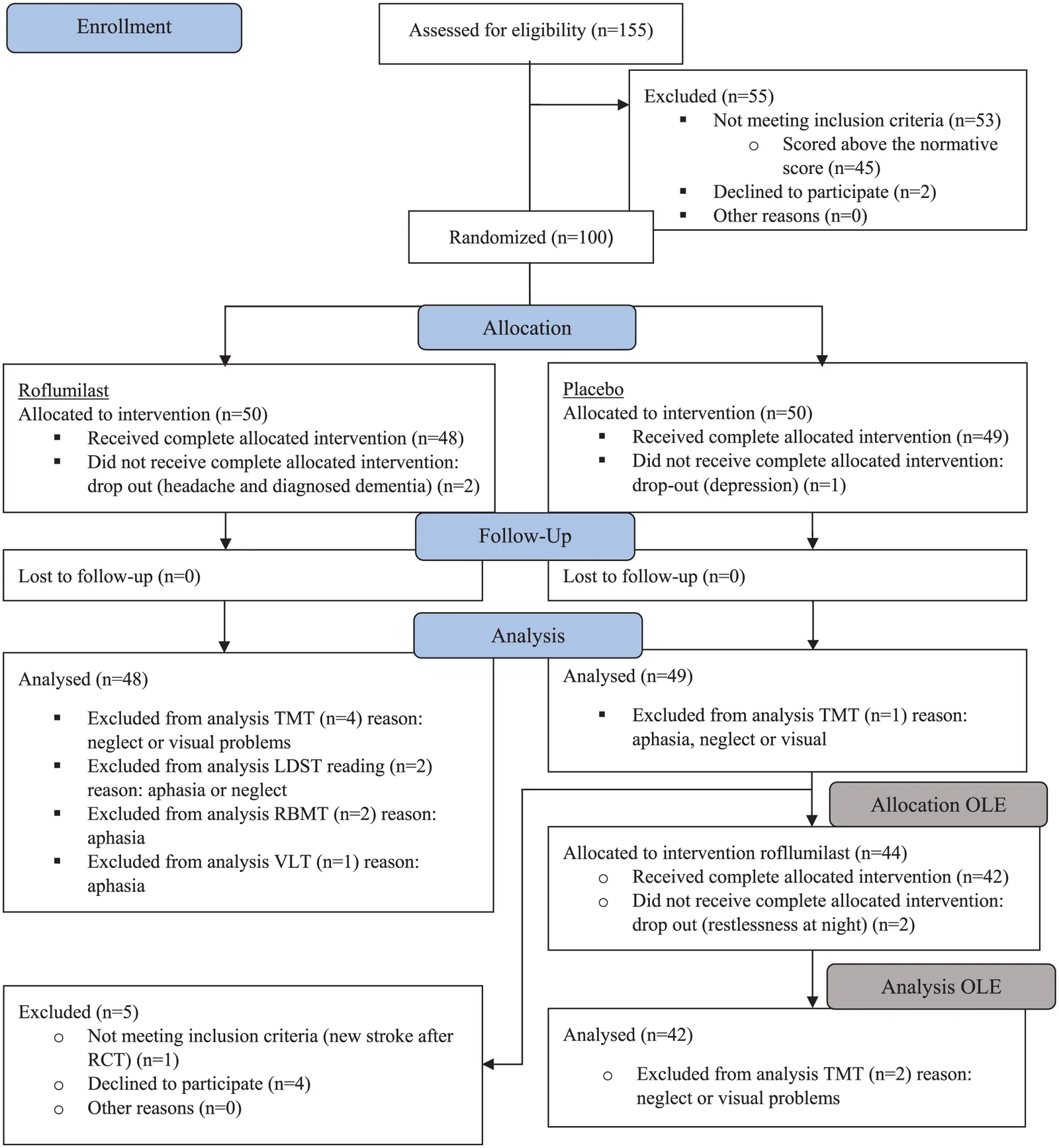
CONSORT flow diagram. CONSORT: Consolidated standards of reporting trials.

Data processing and analysis were performed in SPSS 27.0 and R (version 4.1.1; R Core Team, 2021). Descriptive statistics for continuous parameters included sample sizes, mean, and SD. Descriptive statistics for mean outcome scores included standard error (SE). For categorical data, summary tables present counts and percentages. Data were checked on assumptions of normality, homoscedasticity, multicollinearity, and outliers. There were no significant outliers, other assumptions were fulfilled, and no transformations were necessary. The participants were included in the analysis per protocol; therefore, the drop-out data were not included; the CONSORT flow diagram can be found in Figure 2.

Different statistical methods were employed for the RCT and the OLE phase to appropriately address the distinct study designs and analytical goals. For the RCT, the primary interest was in comparing treatment outcomes between two independent groups (placebo vs. roflumilast) at the study endpoint (T3). Therefore, an analysis of covariance (ANCOVA) was conducted using change-from-baseline scores as the dependent variable, with treatment group as the between-subject factor and baseline measurement of the outcome as an adjusting independent variable. Adjusting for prognostic covariates, and in particular the baseline value of outcomes, is recommended by the EMA and the FDA to increase the statistical power of efficacy analyses and the precision on the treatment effect estimate (European Medicines Agency, 2015; Food and Drug Administration, 2023). This approach allows for a direct interpretation of treatment-related improvements or declines. For the statistical analyses, the VLT and RBMT-stories scores were converted into z-scores. The z-scores are based on norm scores adjusted for age, gender, and education (Schmand et al., 2012; Van der Elst et al., 2005). Age, Gender, and Educational level were included as additional factors for the measures for which no norm scores were available. Their benefit in the analysis model was evaluated by computing the proportion of variance explained (R^2^) when adding these covariates in the model.

In addition to the original protocol, we performed a post hoc exploratory analysis on the main endpoints while adjusting for an additional covariate representing patient’s prognosis to confirm that the observed treatment effect could not be attributed to baseline imbalances between the cohorts. In this analysis, the additional prognostic covariate used was defined a priori using the Placebell methodology (see: Branders et al., 2021, 2022), designed to follow the recommendation of the FDA to leverage a priori knowledge (historical data, literature) to build and use a prognostic index (FDA FG, 2023). This covariate was defined according to the rules and advice suggested by Ooghe et al. (2025) before seeing any data of the current study. It combined the baseline efficacy measurements (standardized results to cognitive tasks including the VLT and RBMT-stories at baseline), demographical information (age, gender, level of education, time since injury), and the available baseline psychological factor (HADS-D scores). As for the primary model, the benefit of this covariate for the analysis model was evaluated by computing the R^2^ when adding this covariate in the model (already adjusted for the baseline value of the outcome). The significance level was set at α = 0.05. A Fisher’s exact test was used to check whether there were statistically significant differences between the two groups in the number of adverse events.

The OLE phase involved repeated assessments within the same participants, all of whom received roflumilast. To evaluate changes over time, instead of the results at the end, a different statistical method was used. To investigate the inter-individual changes, a general linear model for repeated measures was applied, with Time (three levels) as the within-subject factor. The final RCT session score was included as a baseline covariate to control for inter-individual variability at the start of the extension. In cases where outcome measures were not transformed into z-scores, age, gender, and educational level were added as additional covariates to account for potential demographic confounding. This model structure allowed for the examination of within-subject changes across time and improved the precision of estimated treatment effects during the open-label phase.

## Results

For the analysis, some participants were excluded from certain tasks (see Figure 2). Some of the participants had aphasia or problems with reading or vision (e.g. neglect). For these people, certain tasks were not possible to execute or were not valid. For the baseline delayed recall scores of the RBMT-stories, two measurements were missing. An overview of the descriptive characteristics of the population is shown in Table 1. Baseline characteristics (including CVA type) were similar across treatment groups.

**Table 1.** Demographics.

	Roflumilast (*n* = 48)	Placebo (*n* = 49)
Gender		
Female	19 (39.6)	19 (38.8)
Male	29 (60.4)	30 (61.2)
Level of education		
Primary vocational education	3 (6.3)	7 (14.3)
General secondary education	2 (4.2)	2 (4.1)
Secondary vocational education	22 (45.8)	19 (38.8)
Higher general/pre-university secondary education	1 (2.1)	3 (6.1)
Higher professional education	20 (41.7)	13 (26.5)
University	0	5 (10.2)
Type of stroke		
Ischemic stroke	29 (60.4)	33 (67.3)
Hemorrhagic stroke	10 (20.8)	8 (16.3)
TIA	1 (2.1)	4 (8.2)
SAB	2 (4.2)	0 (0)
Brain stem stroke	3 (6.3)	2 (4.1)
>2 of the above	2 (4.2)	1 (2.0)
Stroke type unknown	1 (2.1)	1 (2.0)
Age patient at baseline (years), Mean (±SD), R	60.7 (±6.2), 42–70	59.7 (±7.1)
Time since injury at baseline (years), Mean (±SD), R	5.3 (±4.5), 1–19	4.8 (±4.3)

*Note.* Data are mean (SD) or n (%). For continuous data, the range (R) is included. TIA: Transient ischemic attack; SAB: Subarachnoid bleeding; SD: Standard deviation. All values were not different between the two groups (all tests comparing the groups have a *p*-value >0.467).

### Randomized placebo-controlled trial

One hundred participants were included in the study, of which 50 were in the roflumilast and 50 in the placebo group. After randomization and after the first test day, in total three subjects dropped out. The CONSORT flow diagram is shown in Figure 2. The recruitment took place between July 7, 2021, and March 8, 2023. Inclusion ended when the sample size target was reached.

**Figure 3.**
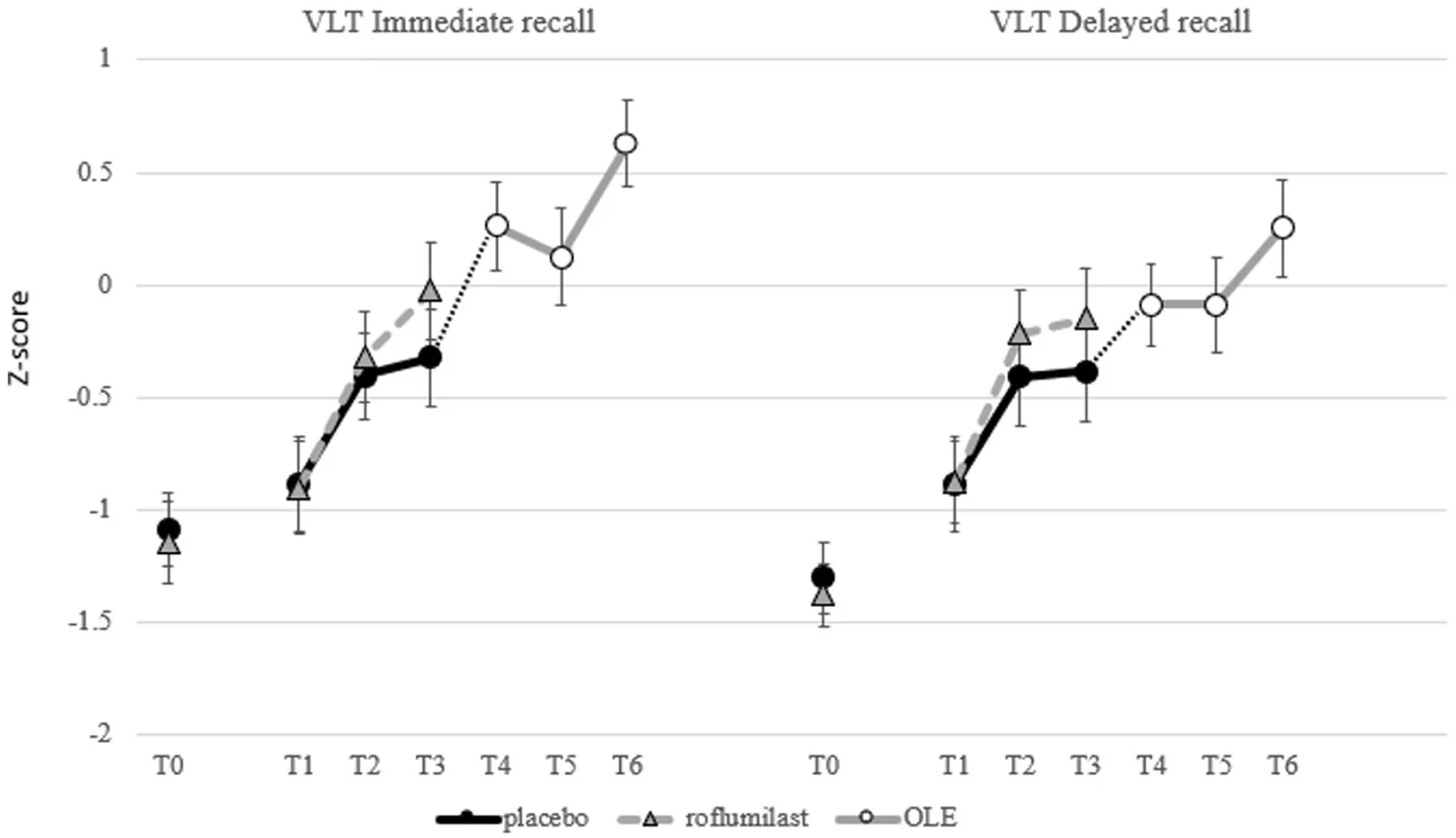
Performance of the placebo and roflumilast group on the VLT immediate and delayed recall z-scores per time point. ***Note.*** T0: baseline assessment; T1: first assessment after acute treatment; T2: assessment after 6 weeks treatment; T3: assessment after 12 weeks treatment, T4: first assessment after acute treatment OLE, T5: assessment after 6 weeks treatment in OLE, T6: assessment after 12 weeks treatment in OLE. Data represent mean and SE. OLE: Open label extension; VLT: Verbal learning test.

**Figure 4.**
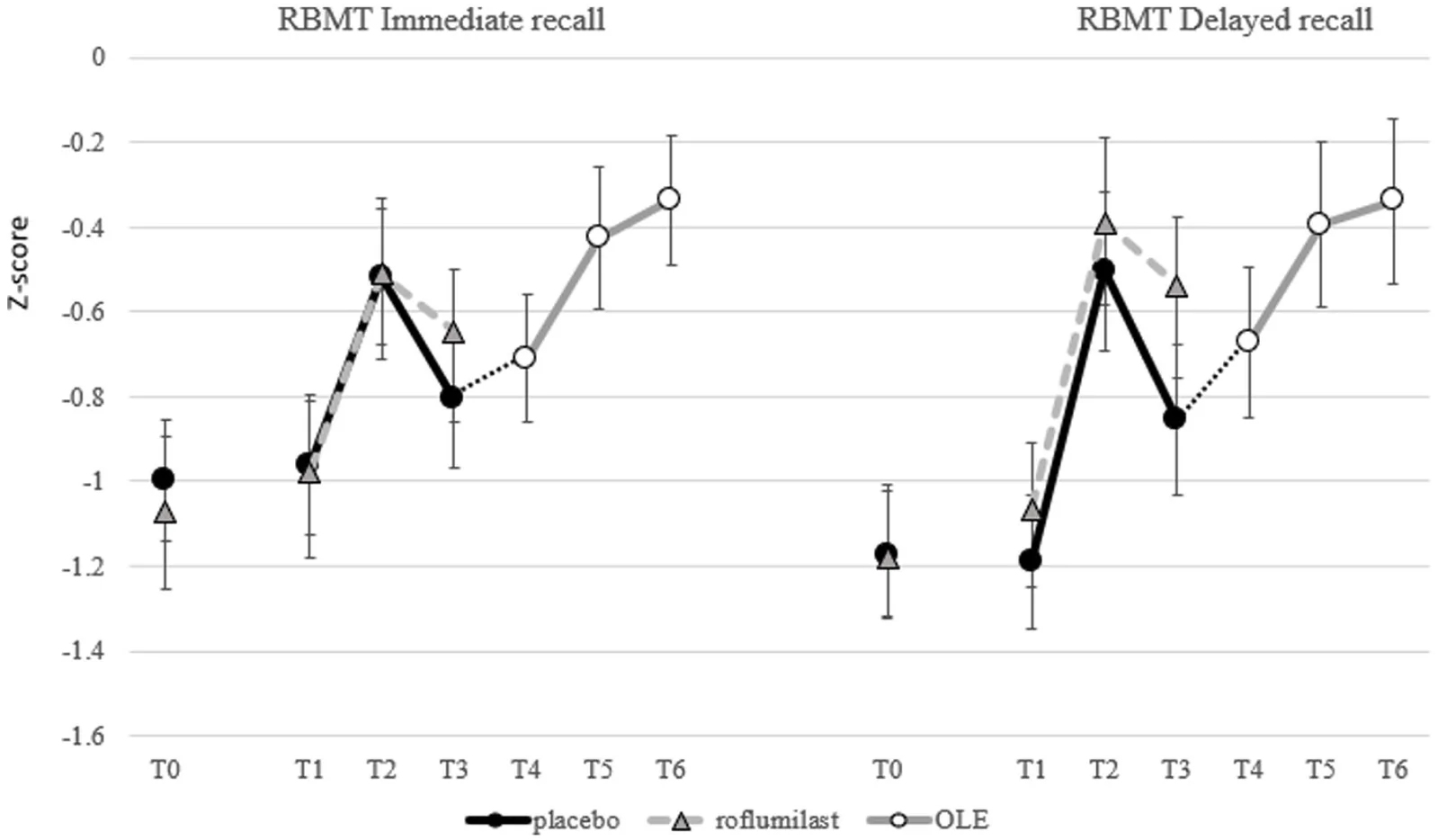
Performance of the placebo and roflumilast group on the RBMT-stories immediate and delayed recall *z*-scores per time point. ***Note.*** T0: baseline assessment; T1: first assessment after acute treatment; T2: assessment after 6 weeks treatment; T3: assessment after 12 weeks treatment, T4: first assessment after acute treatment OLE, T5: assessment after 6 weeks treatment in OLE, T6: assessment after 12 weeks treatment in OLE. Data represent mean and SE. OLE: Open label extension; SE: Standard error; RBMT: Rivermead Behavioural Memory Test.

Table 2 shows the mean changes (and SE) from baseline to endpoint (T3) for each outcome variable, separated by group. Table 2 additionally shows the results of the primary ANCOVA analysis. For all variables except for the normative scores of the VLT and RBMT-stories, the scores were corrected for Gender, Age, and Education. The amount of variance that was explained by these variables is included in Table 2. The primary analysis indicated a consistent pattern of higher performance on the memory tasks (immediate and delayed recall of VLT and RBMT-stories) with roflumilast. This improvement with roflumilast was consistent for all tasks and all time points (see Table 2 and Figures 3 and 4). At 3 months, the treatment effect estimated by these four tasks showed moderate effect sizes (according to Cohen’s classification) with values between 0.31 and 0.36. Although the corresponding p-values did not reach conventional significance thresholds, the direction and magnitude of effects suggest a possible beneficial signal of roflumilast. For the other outcomes, roflumilast did not show any improvement in comparison to placebo.

**Table 2.** Change from baseline to endpoint of primary and secondary outcomes per group, and the treatment effect as assessed with an ANCOVA.

Outcome variables	Placebo		Roflumilast				ANCOVA treatment effect		
	*N*	Mean (SE)	*N*	Mean (SE)	Mean (SE)	Confidence interval	*p*-value	Cohen’s *d*	Variance explained by age, gender, education
VLT immediate	49	0.77 (0.15)	47	1.13 (0.15)	0.36 (0.216)	−0.07, 0.79	0.099	0.34	—
VLT delayed	49	0.91 (0.14)	47	1.26 (0.14)	0.348 (0.196)	−0.04, 0.74	0.079	0.36	—
RBMT-stories immediate	49	0.16 (0.12)	46	0.41 (0.12)	0.25 (0.167)	−0.08, 0.58	0.138	0.31	—
RBMT-stories delayed	47	0.32 (0.13)	46	0.64 (0.13)	0.321 (0.184)	−0.05, 0.69	0.085	0.36	—
TMTA	48	−10.92 (1.87)	44	−8.78 (1.95)	2.143 (2.705)	−3.23, 7.52	0.430	0.17	3.1% (*p* = 0.435)
TMTB	48	−26.74 (3.76)	44	−24.26 (3.93)	2.473 (5.448)	−8.36, 13.30	0.651	0.10	14.9% (*p* = 0.003)
TMTB/A index	48	0.08 (0.12)	44	0.02 (0.13)	−0.056 (0.179)	−0.41, 0.30	0.756	−0.07	8.5% (*p* = 0.053)
LDST writing	49	4.44 (0.82)	46	4.51 (0.85)	0.076 (1.197)	−2.30, 2.46	0.949	0.01	10.6% (*p* = 0.018)
LDST reading	49	4.21 (0.85)	46	5.17 (0.87)	0.96 (1.223)	−1.47, 3.39	0.434	0.16	11.4% (*p* = 0.013)
EMQ	49	−6.22 (1.22)	48	−7.29 (1.24)	−1.074 (1.741)	−4.53, 2.39	0.539	−0.13	4.7% (*p* = 0.220)
USER-P frequency	49	−0.79 (0.82)	48	−0.12 (0.83)	0.667 (1.175)	−1.67, 3.00	0.572	0.12	1.5% (*p* = 0.705)
USER-P restrictions	49	2.56 (1.58)	48	3.68 (1.59)	1.121 (2.243)	−3.34, 5.58	0.619	0.10	7.6% (*p* = 0.065)
USER-P satisfaction	49	0.95 (1.74)	48	2.31 (1.76)	1.361 (2.483)	−3.57, 6.29	0.585	0.11	1.4% (*p* = 0.738)
HADS	49	−0.42 (0.38)	48	−0.93 (0.38)	−0.511 (0.542)	−1.59, 0.57	0.349	−0.19	6.4% (*p* = 0.111)

*Note.* SE: Standard error; VLT: Verbal learning test; RBMT: Rivermead Behavioural Memory Test; TMTA: Trail Making Test Part A; TMTB: Trail Making Test Part B; LDST: Letter-digit substitution test; EMQ: Everyday Memory Questionnaire; USER-P: Utrecht Scale for Evaluation of Rehabilitation-Participation; HADS: Hamilton Anxiety and Depression Scale; ANCOVA: Analysis of covariance.

As shown in Table 3, the treatment effect estimates at T3 after adjustment for the additional prognostic covariate were observed, post hoc, similar in magnitude (Cohen’s *d* between 0.33 and 0.40) and now reached significance thresholds (VLT delayed recall, *p* = 0.044 and RBMT-stories delayed recall, *p* = 0.036). The post hoc exploratory prognostic adjusted analyses reduced the variance in the analysis models for VLT immediate and delayed, and RBMT-stories immediate and delayed, by 8.7%, 11.2%, 12.0%, and 15.0%, respectively, which improved the precision on the treatment effect estimates. Besides, the similarity of the treatment effect in the prognostic-adjusted and non-adjusted analyses suggests that this observed effect is not due to any baseline imbalance between the cohorts and strengthens the confidence in study results.

**Table 3.** Change from baseline to endpoint of memory performance (VLT and RBMT-stories) adjusted for the additional prognostic covariate per group and the treatment effects as assessed with an ANCOVA.

Outcome variables	Placebo		Roflumilast			ANCOVA treatment effect			
	*N*	Mean (SE)	*N*	Mean (SE)	Mean (SE)	Confidence interval	*p*-value	Cohen’s *d*	Variance explained by the prognostic covariate
VLT immediate	49	0.75 (0.15)	47	1.15 (0.15)	0.397 (0.208)	−0.02, 0.81	0.059	0.38	8.7% **(***p* = **0.004)**
VLT delayed	49	0.89 (0.13)	47	1.28 (0.13)	0.38 (0.186)	0.01, 0.75	0.044	0.40	11.2% **(*p*** < **0.001)**
RBMT-stories Immediate	49	0.15 (0.11)	46	0.42 (0.11)	0.268 (0.158)	−0.05, 0.58	0.093	0.33	12.0% **(***p* < *0*.**001)**
RBMT-stories delayed	47	0.3 (0.12)	46	0.66 (0.12)	0.364 (0.171)	0.02, 0.70	**0.036**	0.41	15.0% **(***p* < **0.001)**

*Note.*, SE: Standard error; VLT: Verbal learning test; RBMT: Rivermead Behavioural Memory Test; ANCOVA: Analysis of covariance. Bold values indicate *p*-values < 0.05.

### Open label extension

At the end of the RCT, participants were unblinded and the placebo group was asked whether they wanted to participate in the OLE. In this part of the study, the participants were aware that they now received roflumilast. Of the 49 participants who completed the RCT, 4 were not willing to participate in the OLE study. One person did not fulfill the inclusion criteria, since they had a new CVA between the end of the RCT and the start of the OLE. Forty-four participants started the OLE study.

The total scores per measurement and the outcomes of the primary and secondary analyses are shown in Table 4. The data showed a significant increase over time with medium to large effects sizes in the number of items recalled during the immediate (Partial η^2^: 0.171) and delayed recall (Partial η^2^: 0.079) of the VLT (see Figure 3). For all variables of the RBMT-stories, immediate (Partial η^2^: 0.115) and delayed recall (Partial η^2^: 0.095) scores increased significantly over time with medium to large effect sizes (see Figure 4). Additionally, a significant effect was found on the LDST reading (Partial η^2^: 0.08).

**Table 4.** Results of general linear model for all primary and secondary outcome measures.

Outcome variables	*N*	BL^a^	T4	T5	T6	*F*-test _(df, df)_ (*p*) Time	Interaction variable × Time	Partial η^2^
VLT Immediate	42	−0.34 (0.23)	0.26 (0.21)	0.13 (0.23)	0.63 (0.21)	F_(2, 82)_ = 8.475 **(***p* ⩽ 0.001**)**	—	0.171
VLT delayed	42	−0.30 (0.23)	−0.09 (0.20)	−0.09 (0.23)	0.25 (0.23)	F_(2, 82)_ = 3.520 (*p* = **0.034)**	—	0.079
RBMT-stories immediate	42	−0.88 (0.16)	−0.72 (0.16)	−0.42 (0.18)	−0.36 (0.16)	F_(2, 82)_ = 5.319 **(*p* = 0.007)**	—	0.115
RBMT-stories delayed	42	−0.88 (0.18)	−0.67 (0.19)	−0.39 (0.21)	−0.34 (0.21)	F_(2, 82)_ = 4.320 **(*p* = 0.016)**	—	0.095
TMTA	40	34.8 (2.04)	34.2 (2.39)	32.9 (2.24)	32.0 (1.95)	F_(2, 78)_ = 1.406 (*p* = 0.251)	—	0.035
TMTB	40	87.3 (6.32)	82.1 (6.34)	82.0 (6.17)	84.2 (8.14)	F_(2, 76)_ = 0.114 (*p* = 0.114)	Time × baseline: F_(2,76)_ = 3.300, **(*p* = 0.042)**	0.080
TMTB/A index	40	2.6 (0.13)	2.5 (0.13)	2.5 (0.11)	2.6 (0.17)	F_(2, 76)_ = 3.780 **(*p* = 0.027)**	Time × baseline: F_(2,76)_ = 5.114, **(*p* = 0.008)**	0.119
LDST writing	42	42.8 (1.78)	43.6 (1.82)	44.2 (1.87)	45.1 (1.88)	F_(2, 82)_ = 2.491 (*p* = 0.089)	—	0.057
LDST reading	42	48.4 (1.96)	49.1 (2.04)	50.1 (2.18)	51.1 (2.11)	F_(2, 82)_ = 3.506 **(*p* = 0.035)**	—	0.079
EMQ	42	22.0 (1.73)	21.3 (1.96)	19.5 (1.91)	17.4 (1.73)	F_(2, 80)_ = 19.702 **(*p* ⩽ 0.001)**	Time × Gender: F_(2, 80)_ = 14.395, **(*p* ⩽ 0.001)**	0.265
USER-P frequency	42	36.7 (1.23)	36.7 (1.13)	37.3 (1.08)	36.2 (1.26)	F_(2, 82)_ = 0.619 (*p* = 0.541)	—	0.015
USER-P restrictions	42	80.8 (2.30)	81.9 (2.41)	81.8 (2.58)	80.3 (2.47)	F_(2, 82)_ = 1.026 (*p* = 0.363)	—	0.024
USER-P satisfaction	42	68.7 (2.45)	70.0 (2.36)	68.9 (2.28)	67.7 (2.11)	F_(2, 80)_ = 4.224 **(*p* = 0.018)**	Time × Gender: F_(2, 80)_ = 5.110, **(*p* = 0.008)**	0.113
HADS-NL	42	3.2 (0.45)	3.9 (0.42)	3.7 (0.45)	4.0 (0.52)	F_(2, 82)_ = 0.197 (*p* = 0.821)	—	0.005

*Note.* VLT: Verbal learning test; RBMT: Rivermead Behavioural Memory Test; TMTA: Trail Making Test Part A; TMTB: Trail Making Test Part B; LDST: Letter-digit substitution test; EMQ: Everyday Memory Questionnaire; USER-P: Utrecht Scale for Evaluation of Rehabilitation-Participation; HADS: Hamilton Anxiety and Depression Scale.

a T3 is the final measurement of the double-blind RCT, T4 is an hour after first drug intake, T5 is 6 weeks after the start of treatment and T6 is 12 weeks after start. Data are shown as mean (standard error). Bold values indicate *p*-values < 0.05.

**Figure 5.**
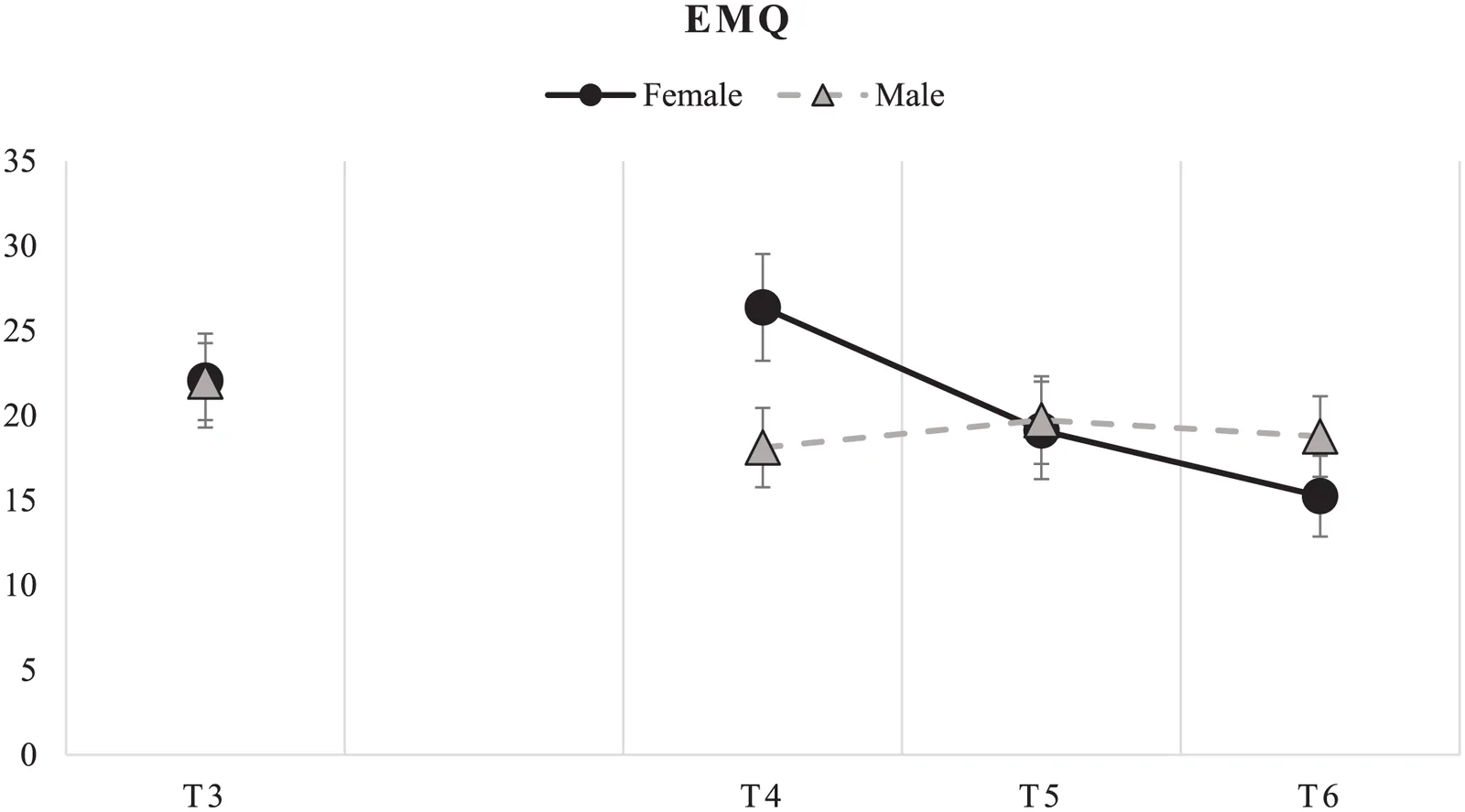
Mean and standard error EMQ scores per gender. ***Note.*** T3: assessment after 12 weeks treatment, T4: first assessment after acute treatment OLE, T5: assessment after 6 weeks treatment in OLE, T6: assessment after 12 weeks treatment in OLE. OLE: Open label extension; EMQ: Everyday Memory Questionnaire.

For all analyses, interaction effects were analyzed for baseline performance, and for Gender, Age, and Education. The interaction effects of Gender, Age, and Education were not analyzed for the normative scores of the VLT and RBMT-stories, since the scores are already corrected for these variables. The covariates were included in a stepwise backward elimination manner. Significant interaction effects are reported in Table 4. For the TMTB and TMTB/A index, a significant interaction effect was found for baseline scores on time, indicating that the level of baseline performance had a significant effect on how the performance progressed over time. For the EMQ, an interaction effect of Gender on Time was found; this was further investigated by comparing the means and doing the analysis per gender. This analysis indicated that the score on the EMQ only significantly decreased over time in females. Thus, their subjective complaints decreased. For results on these analyses, see Table 5 and Figure 5. For the USER-P satisfaction, an interaction effect of Gender on Time was found as well. The raw data showed inconsistent fluctuations within the groups, and the analyses separate per Gender were not significant. For mean scores and analyses, see Table 5.

**Table 5.** Mean and standard deviations separate for gender for EMQ and USER-P satisfaction.

Outcome variables	Gender	*N*	BL^a^	T4	T5	T6	*F*-test_(df, df)_ (*p*) Time
EMQ	Female	16	22.1 (2.78)	26.4 (3.15)	19.1 (2.88)	15.3 (2.38)	F_(2, 30)_ = 13.585 **(*p*** **⩽** **0.001)**
	Male	26	22.0 (2.28)	18.1 (2.33)	19.7 (2.57)	18.8 (2.37)	F_(2, 50)_ = 0.908 (*p* = 0.410)
USER-P satisfaction	Female	16	68.7 (3.70)	70.5 (3.88)	64.4 (3.03)	67.2 (2.93)	F_(2, 30)_ = 3.214 (*p* = 0.054)
	Male	26	68.7 (3.31)	69.6 (3.02)	71.6 (3.08)	68.1 (2.96)	F_(2, 50)_ = 2.862 (*p* = 0.067)

*Note.* Data are shown as mean (standard error). EMQ: Everyday Memory Questionnaire; USER-P: Utrecht Scale for Evaluation of Rehabilitation-Participation.

a T3 is the final measurement of the double-blind RCT, T4 is an hour after first drug intake, T5 is 6 weeks after the start of treatment, and T6 is 12 weeks after start. Bold values indicate *p*-values < 0.05.

### Registered side effects

In total, three subjects dropped out of the RCT phase of the study. In the roflumilast group, one male subject received the diagnosis of dementia (the participant did not conceal information about possible mild dementia diagnosis at the medical screening) and did no longer fulfill the inclusion criteria, and one female subject did not want to participate anymore since she experienced headaches as a possible side effect. In the placebo group, one female subject had a relapse of a depressive episode and no longer fulfilled the inclusion criteria. In the OLE phase, two participants dropped out: the first due to restlessness at night/insomnia (after 29 days), and the second due to insomnia and nausea (after 10 days). Thus, there was a 4.6% (2 out of 44) drop-out due to restlessness/insomnia and a 2.3% (1 out of 44) drop-out due to nausea. All medical problems or physical complaints the participants had during the trial were registered in their medication diaries and discussed with and signed by a medical doctor. Unfortunately, although participants were asked to classify complaints as mild, moderate, or severe, most did not provide this information. Serious complaints that led to a doctor’s appointment were registered as SAE. An overview of all AEs and results of Fisher’s exact tests between the groups can be found in Table 6. The incidence of the AEs was similar in both treatment groups. The SAEs are shown in Table 7. There were no SAEs in the OLE phase.

**Table 6.** A summary of the most frequently reported adverse events (occurrence > 1).

Adverse symptoms	Placebo (*n* = 49)	Roflumilast (*n* = 48)	*p*-value	OLE (*n* = 44)
Nervous system				
Headache	11 (22)	7 (15)	0.434	4 (10)
Dizziness	5 (10)	6 (13)	0.759	3 (7)
Gastrointestinal problems				
Diarrhea	1 (2)	4 (8)	0.204	4 (10)
Nausea	3 (6)	3 (6)	1	1 (2)
Stomach ache	2 (4)	1 (2)	1	1 (2)
Metabolism and nutrition				
Reduced appetite	2 (4)	—	0.495	—
Increased weight	2 (4)	—	.495	—
Sleep/Fatigue				
Insomnia/restlessness at night				2 (5)
Fatigue	3 (6)	7 (15)	0.199	3 (7)
Vascular problems				
Palpitations	—	—	—	2 (5)
High blood pressure	—	—	—	2 (5)
Cognitive				
Memory loss	3 (6)	—	0.242	2 (5)
Musculoskeletal problems				
Muscle pain	3 (6)	—	0.242	—
Pain in limbs or joints	1 (2)	3 (6)	0.362	1 (2)
Back/neck pain	—	3 (6)	0.243	2 (5)
Infections				
Flue/ coughing/ ill	7 (14)	6 (13)	0.769	9 (21)

*Note.* Data are reported as *N* (%). OLE: Open label extension.

**Table 7.** An overview of the SAEs in the RCT.

	When	Group
Recurrent stroke	5 days after end of treatment	Placebo
Epileptic attack (previous epileptic attack before participation)	8 weeks after end of treatment	Roflumilast
Episode of previously diagnosed conversion disorder	4 weeks after end of treatment	Roflumilast

*Note.* There were no SAEs in the OLE phase. OLE: Open label extension; RCT: Randomized placebo-controlled trial; SAE: serious adverse events.

## Discussion

The present RCT and subsequent OLE investigated the potential of a PDE4 inhibitor for treating cognitive impairments following CVA. The primary hypothesis was that roflumilast would enhance episodic memory, as assessed by the delayed recall component of the VLT. Results indicated a trend toward improved memory performance in the roflumilast group compared to placebo, with between-group differences emerging after 3 months of treatment in the RCT phase. These differences were associated with moderate effect sizes. A post hoc exploratory analysis, leveraging an informative and predefined covariate representing the prognosis of the patients, showed similar effect sizes and a significant treatment effect for all delayed tasks (VLT and RBMT-stories). In the subsequent OLE, where participants were unblinded for 3 months of treatment with roflumilast, the memory performance in both immediate and delayed tasks (VLT and RBMT-stories) significantly improved. Interestingly, this study showed medium effect sizes for delayed recall and large effect sizes for immediate recall. Additionally, reduced subjective memory complaints (EMQ scores) were found only for women. In this study, however, the limitations of the design have to be taken into account, especially possible placebo-effect enhanced by unblinding. Other limitations will be discussed below. These findings suggest that roflumilast has potential to improve episodic memory in patients with PSCI.

In the RCT study, the effects of roflumilast appeared to be limited to memory tasks since the other tasks did not reveal treatment effects. This is in line with previous research in which improvement in VLT performance was found in healthy young and old humans (Blokland et al., 2019b; Van Duinen et al., 2018). Furthermore, this is what would be expected based on preclinical data showing that PDE4 inhibitors were found to reverse brain-injury induced memory deficits (Blokland et al., 2019a; Reneerkens et al., 2009; Schreiber et al., 2020; Soares et al., 2015; Vilhena et al., 2021; Wilson et al., 2016). Additionally, it is in line with a study that showed roflumilast’s potential to improve verbal memory function in schizophrenia patients (Gilleen et al., 2021). The fact that we find the effects of roflumilast in memory tasks could be explained considering that in brain injury models, the levels of PDE4 are upregulated in the hippocampus (Wilson et al., 2016). By inhibiting PDE4 with roflumilast, it can be expected that this will have a positive effect on neuroplasticity in the hippocampus and thus memory performance (Schreiber et al., 2020).

There was an unexpected substantial improvement in immediate and delayed VLT performance in the placebo group from T0 to T2 in the RCT. This could be related to a training effect on the VLT task, which has been reported for the VLT (Van Der Elst et al., 2008). These effects were apparent until T2, after which no further improvement was visible in the placebo group (see Figure 3). In addition, the initial improvement could also be explained by a placebo response in both groups. It is known that motivation and emotion can influence cognition (Madan, 2017). The patients in this study, for whom no treatment exists, were very enthusiastic and grateful to participate in this study that could treat their cognitive problems. This probably created high expectations and made them extra motivated to perform the tasks. To control for baseline variations that could have affected the performance in the VLT, a post hoc exploratory ANCOVA using prognostic covariates was conducted (Branders et al., 2021, 2022). This resulted in similar treatment effects, with the prognostic covariates explaining more variance and appearing to reduce the placebo effect, leading to a statistically significant difference between the placebo and roflumilast groups. We did not control for these placebo-effects in the OLE study. Therefore, these results should be interpreted with care and can only be seen as limited proof.

In the OLE study, the training effects were less likely to occur since the participants had already been tested four times on the test battery. Therefore, enhanced performance on the memory tasks is likely attributable to either the treatment or a placebo effect. For the RBMT-stories, it should be taken into account that only two alternating parallel test versions were used. Thus, the same versions were repeated every other time. Therefore, training effects could still be seen on the RBMT-stories outcomes. Consequently, these results should be interpreted with care. Interestingly, the performance was improved in the VLT and RBMT-stories tasks, which both are related to improved episodic memory. These findings further support the notion that PDE4 inhibition specifically improves episodic memory. The improved performance in the LDST reading task suggests an additional effect of roflumilast on other cognitive functions (e.g. processing speed). No effects were found in the TMT and the LDST writing. While these findings may argue against a global practice effect, the presence of a placebo-related effect cannot be definitively dismissed.

As mentioned, it cannot be excluded that higher motivation in these patients, who now knew that they received roflumilast, may have had some effect on the performance in the memory tasks. This interpretation is supported by the observation that mean scores at the start of the OLE were higher than those at the final assessment of the RCT. However, this early improvement may also reflect acute pharmacological effects of roflumilast, as reported previously (Blokland et al., 2019b). A purely placebo-related explanation seems less likely, given that a clear improvement in performance was observed toward the end of the study (from T5 to T6). Placebo effects typically occur shortly after treatment initiation and would, therefore, be more expected between T3 and T4 than at later time points.

The smaller gains observed from T4 to T6, compared with the larger improvements from T1 to T3 in the placebo group, can be explained by two factors. First, stronger practice effects are expected during the T0–T3 phase, when participants were still unfamiliar with the cognitive tasks, whereas during the OLE phase participants had already completed the tasks multiple times, making further training effects unlikely. Second, by the end of the RCT, performance of the placebo group had already approached normative levels (z-scores of almost 0, corrected for age, sex, and education), leaving less room for further improvement during the OLE. Together, reduced practice effects and a ceiling effect provide an explanation for the smaller gains observed from T4 to T6.

The temporal pattern and cognitive profile of the treatment effects observed in the present study differ from those reported in earlier work on PDE4 inhibition. Whereas we observed a roflumilast effect only after 12 weeks of treatment, previous studies have reported improvements after substantially shorter exposure periods, within hours in young healthy adults (Van Duinen et al., 2018) or after approximately 8 days in patients with schizophrenia (Gilleen et al., 2021). Several factors may account for these discrepancies. First, earlier studies predominantly employed placebo-controlled, cross-over, within-subject designs, which effectively control for training, practice, and placebo effects associated with repeated cognitive testing. In contrast, the between-subjects design of the current study may have allowed such effects to mask subtle treatment-related differences, particularly during the earlier phases of the intervention. In addition, our participants were chronic stroke patients with persistent and substantial cognitive deficits, as reflected by markedly reduced baseline z-scores. Compared with the populations studied previously, this group is likely more severely affected at the cognitive and neurobiological level, making it reasonable to expect that pharmacological enhancement requires a longer treatment duration before producing detectable behavioral effects. Over the extended 12-week testing period, practice-related improvements may initially obscure treatment effects, which then become apparent only after sufficient cumulative exposure to roflumilast.

With respect to the distinction between immediate and delayed memory, previous studies have reported mixed findings following PDE4 inhibition. Gilleen et al. (2021) observed their strongest effects on immediate verbal learning (i.e. the third learning trial of the VLT), whereas Blokland et al. (2019b)reported enhancement primarily on delayed recall, and Van Duinen et al. (2018) found effects on immediate recall in young healthy adults. Notably, these studies investigated subchronic/acute treatment effects, whereas the current study examined a prolonged 12-week intervention. In the RCT, medium effect sizes were observed for both immediate and delayed recall on the VLT and RBMT stories, whereas in the OLE, large effects were observed for immediate recall and medium effects for delayed recall. Taken together, these findings suggest that, across study phases, the cognitive effects of roflumilast appear overall more robust for immediate recall than for delayed recall. In addition, the delayed emergence of treatment effects in the present study likely reflects both population characteristics, for example, differences in pathology and PDE4 levels, and methodological factors, as discussed before, related to repeated testing over time.

With regard to clinical relevance, it is important to interpret the observed changes in VLT performance in the context of the study population. Participants were chronic stroke patients with substantial and persistent cognitive impairment, as reflected by markedly low baseline z-scores. Although the VLT is a laboratory test that may not directly translate into daily memory performance, the VLT has been used as a clinical tool to detect and predict memory problems (Green et al., 2011; Hamel et al., 2015; Silva et al., 2012). This indicates that the VLT does reflect aspects of memory problems that occur in daily life. At the end of the RCT, the z-scores were close to zero which would indicate that these participants would no longer be diagnosed as having a memory problem. Moreover, the performance after the OLE was even above the expected score (based on age, sex, and education). Taken together, the improvements in VLT task performance are indicative of a clinically relevant effect on cognition.

Another important point to discuss with regard to clinical relevance is that in this study, we included patients with various types of CVA. This was done because it was assumed that the mechanism to improve memory is independent of CVA type. Thus, the location of the lesion, time since injury, and severity of the injury varied among participants. Lesion location appears to be an important determinant of PSCI (Weaver et al., 2021; Zhao et al., 2018). Zhao et al. (2018) identified a strategic network involving several overlapping and domain-specific cortical and subcortical structures for each of the cognitive domains. Weaver et al. (2021) created a brain map of strategic infarct locations predicting PSCI. This might help clinicians understand and predict the cognitive impact of the CVA (Zhao et al., 2018). Understanding this mapping could also be of importance for interpreting the individual pharmacological effects of a drug such as roflumilast and understanding who will benefit most from this treatment. Ad hoc analyses of the different groups of CVA types (e.g. ischemic/hemorrhagic) did not reveal different outcomes. However, it should be noted that the number of patients per sub-group was too small to draw such a conclusion. Additionally, we included time since injury as a covariate in the analysis. However, adding this factor as a covariate in the analysis did not have a significant impact.

A possible limitation of the study was the fact that the tests were done at the participants’ homes. Many factors could not be regulated or controlled (e.g. sounds made by doorbells, family members, pets), resulting in distraction during the neuropsychological tests. It could also be seen as a strength since this made the testing more ecologically valid. Additionally, throughout the 3 months, personal factors such as sleep, health, mourning, mood, or social difficulties due to the COVID-19 pandemic (38 participants were tested during the pandemic) also varied among participants. This may have influenced the scores on the questionnaires (EMQ, USER-P, and HADS). An ad hoc analysis did not reveal differences between COVID and non-COVID participants, but this analysis may have been underpowered to make a reliable conclusion.

Several recommendations can be proposed for future research investigating cognitive-enhancing pharmacological interventions in PSCI. While the current study demonstrated preliminary indications of treatment-related improvements in memory, these effects were not consistently reflected in subjective questionnaire responses. An exception was observed in the OLE, where female participants reported improvements in perceived memory performance. It is important to consider that self-awareness may be impaired in individuals with brain injury, potentially limiting their ability to detect cognitive changes in everyday functioning (Jaywant et al., 2022; Terneusen et al., 2022). Therefore, it would be recommended to add a measurement on daily functioning to be filled out by the spouse/caretaker.

With respect to the dose used and the duration of treatment, two recommendations could be suggested. Based on pharmacokinetic data (Prickaerts et al., 2024), it could be suggested to use a lower dose in chronic studies. Modeling plasma levels following acute and chronic treatment with roflumilast suggests that a single acute dose of 100 µg, which has been shown to improve memory in previous studies (Blokland et al., 2019b; Van Duinen et al., 2018), produces plasma concentrations comparable to those achieved with chronic administration of 50 µg. Furthermore, the current findings appear to indicate that the cognitive improvement in PSCI patients started to diverge from the placebo group after 3 months of treatment. A longer treatment duration could have further substantiated this improvement.

Finally, a placebo run-in phase could be included to neutralize the strong placebo response and possible practice effects. By run-in phase, we refer to a brief period in which participants complete one or more assessment sessions before treatment is initiated, allowing performance to stabilize without pharmacological influence. Without such a phase, early performance gains may reflect task-learning rather than treatment-specific effects. Including such a procedure in future studies would help minimize learning/practice effects at the start of the trial and yield a more accurate estimate of true treatment-related change.

This is the first study in which chronic roflumilast treatment (100 µg q.d. for 3 months) was applied, and it showed the potential to improve memory performance in chronic CVA patients. No safety or tolerability issues were observed. These findings encourage further studies with an optimized study design, for example, placebo run-in, and treatment regimen, for example, 6 months, which may provide additional evidence for the use of roflumilast as a promising treatment for memory deficits in stroke patients.

## Supplemental Material

sj-docx-1-jop-10.1177_02698811261436581 – Supplemental material for The effects of the PDE4 inhibitor roflumilast on cognitive performance after a cerebrovascular accident: a double-blind randomized placebo-controlled trial with an open label extension (ROSTMEMA) Supplemental material, sj-docx-1-jop-10.1177_02698811261436581 for The effects of the PDE4 inhibitor roflumilast on cognitive performance after a cerebrovascular accident: a double-blind randomized placebo-controlled trial with an open label extension (ROSTMEMA) by Jill Kerckhoffs, Ieke Winkens, Arthur Ooghe, Samuel Branders, Jos Prickaerts and Arjan Blokland in Journal of Psychopharmacology
