# Supplementary material for: The effects of the PDE4 inhibitor roflumilast on cognitive performance after a cerebrovascular accident: a double-blind randomized placebo-controlled trial with an open label extension (ROSTMEMA)
Source: J Psychopharmacol. 2026 Apr 24;40(9):1415–29. doi: 10.1177/02698811261436581 (PMC13490669; doi:10.1177/02698811261436581)
Supplement: Supplementary file 1 [file sj-docx-1-jop-10.1177_02698811261436581.docx]

**Contents**

[**Supplementary files**	0](#_Toc206755214)

[**A. Overview exclusion criteria**	1](#_Toc206755215)

[**B. Overview of measures at each time point**	2](#_Toc206755216)

## **A. Overview exclusion criteria**

Table S1. Flow chart study

| **Exclusion criterion** | **Reason** |
| --- | --- |
| Normal Pressure Hydrocephalus, Huntington and Parkinson’s disease | Neurodegenerative diseases with different cognitive profiles |
| HIV/AIDS and Hepatitis C & B | Risk factor for physicians: blood withdrawal |
| recent Transient Ischemic Attack (TIA) (< 1 year) | Not in chronic phase |
| COPD Gold Type 3 and 4 | Fewer disease complaints due to the effect of roflumilast and possible intake of roflumilast as medication. |
| History of schizophrenia, bipolar disorder or psychotic symptoms not otherwise specified or previous treatment for these diseases (lifetime), Current affective disorder (i.e. anxiety or major depression) diagnosed by a clinician | Impact on cognitive abilities |
| History of alcohol abuse, brain tumor, encephalitis or epilepsy prior to stroke | Only include cognitive problems due to stroke |
| Current treatment with (or illicit use of) cannabis, opiates, benzodiazepines, MDMA, and cocaine | Impact on cognition |
| Moderate or major liver impairments (e.g. Child-Pugh B and C), use of medication showing strong inhibition of either CYP3A4 or CYP1A2 | Impact absorption of the drug |
| Rare hereditary problems of galactose intolerance, Lapp lactase deficiency, or glucose-galactose malabsorption | Lactose monohydrate in the medication |
|  |  |
|  |  |
| Participation in other drug studies | Confounding results |
| **Changed after amendment** | **Reason** |
| The use of centrally acting beta blockers as exclusion criterion | If patients have a stable dose for at least three months changes in cognition are minimal. Many stroke patients take this medication. |
| COPD Gold Type 1 or 2 as exclusion criterion | Since these patients will not take any drugs for their COPD and will not have benefit on their disease complaints. |
| BMI 18.5-30 | There was no correlation between general health as measured by the medical doctor and BMI, there is no reason to assume the BMI will have an effect on general effect of the drug. |

*Note*. COPD= Chronic Obstructive Pulmonary Disease;

## **B. Overview of measures at each time point**

Table S2. Overview of measures at each timepoint

| **Item** | **Screening** | **T0** | **T1** | **T2** | **T3** | **T4** | **T5** | **T6** |
| --- | --- | --- | --- | --- | --- | --- | --- | --- |
| **Screening** |  |  |  |  |  |  |  |  |
| Informed consent |  | X |  |  |  |  |  |  |
| Demography (e.g. date of birth, Gender, ethnic group) |  | X |  |  |  |  |  |  |
| Psychosocial information (education level) |  | X |  |  |  |  |  |  |
| Medical/psychiatric/surgical history |  | X |  |  |  |  |  |  |
| Blood alcohol level |  |  | X | X | X | X | X | X |
| Final check inclusion/exclusion criteria |  | X |  |  |  |  |  |  |
| **Randomization** |  |  | X |  |  |  |  |  |
| **Efficacy** |  |  |  |  |  |  |  |  |
| Neuropsychological tests |  | X | X | X | X | X | X | X |
| Questionnaires: Subjective memory, Participation, and Mood |  | X | X | X | X | X | X | X |
| **Safety** |  |  |  |  |  |  |  |  |
| Adverse events |  |  |  | X | X | X | X | X |
| Physical examination | X |  |  |  |  |  |  |  |
| Vena puncture | X |  |  |  |  |  |  |  |
| Blood pressure | X | X | X | X | X | X | X | X |
| ECG | X |  |  |  |  |  |  |  |
| **Study drug and other medications** |  |  |  |  |  |  |  |  |
| Previous/concomitant medication | X | X | X | X | X | X | X | X |
| Study medication administration record |  |  |  | X | X |  | X | X |
| Drug Return |  |  |  | X | X |  | X | X |
